# Chikungunya chronic arthralgia: impact on general and mental health and absenteeism from work

**DOI:** 10.1590/0037-8682-0149-2024

**Published:** 2024-12-16

**Authors:** Lorena Gomes Santos, Leile Camila Jacob-Nascimento, Rosângela Oliveira Anjos, Moyra Machado Portilho, Viviane Machicado Cavalcante, Adriane Souza Paz, Mittermayer Barreto Santiago, Cristiane Wanderley Cardoso, Mitermayer Galvão Reis, Guilherme Sousa Ribeiro

**Affiliations:** 1 Universidade Federal da Bahia, Faculdade de Medicina da Bahia, Salvador, BA, Brasil.; 2 Fundação Oswaldo Cruz, Instituto Gonçalo Moniz, Salvador, BA, Brasil.; 3 Escola Bahiana de Medicina e Saúde Pública, Salvador, BA, Brasil.; 4 Secretaria Municipal de Saúde de Salvador, Centro de Informações Estratégicas em Vigilância em Saúde, Salvador, BA, Brasil.

**Keywords:** Chikungunya, Arthralgia, Health assessment, Mental Health, Work Absenteeism

## Abstract

**Background:**

This study investigated the self-rated general health, mental health, and work absenteeism among patients with laboratory-confirmed chikungunya.

**Methods:**

Telephone interviews were conducted with 63 patients ≥22 months after infection.

**Results:**

Patients who reported (N=42) or did not report (N=21) chronic arthralgia, defined by duration ≥90 days, had different frequencies for low scores for general health (68.3% vs. 30.0%, respectively; prevalence ratio, 95% confidence interval: 2.3, 1.1-4.6), symptoms of depression (31.7% vs. 15.0%; 2.1, 0.7-6.6), symptoms of anxiety (43.9% vs. 35.0%; 1.3, 0.6-2.5), and work absenteeism (76.5% and 40.0%; 1.9, 0.9-4.2).

**Conclusions:**

Chikungunya chronic arthralgia impacts long-term health and work.

Chikungunya typically manifests as fever and arthralgia of acute onset, and the latter can persist for months to years in more than 40% of patients^1^, potentially affecting daily activities^2^. Although previous studies have described the physical impact, mental suffering, reduced quality of life, absenteeism from work, and other disabilities associated with chikungunya chronic arthralgia^3^ ^,^ ^4^, studies comparing these outcomes between patients with laboratory-confirmed chikungunya who have and have not developed persistent arthralgia are less common^5^ ^,^ ^6^. Moreover, studies evaluating the health impacts of chronic chikungunya in different endemic areas are needed to assess whether differences between populations and chikungunya virus (CHIKV) strains influence the observed effects of CHIKV infection. This study aimed to describe the self-rated general health, mental health, and work absenteeism of patients with chikungunya in Brazil, comparing those with and without persistent arthralgia following acute disease.

Between October 2021 and January 2022, we interviewed, by telephone, adult patients (≥ 18 years of age) with a previous laboratory diagnosis of chikungunya to obtain data on the persistence of symptoms, mental health, quality of life, and work absenteeism. These chikungunya patients were identified during a surveillance study that investigated arbovirus infections among patients seeking healthcare for an acute febrile or exanthematous illness at a public outpatient emergency unit in Salvador, Brazil, between June 2019 and March 2020. The laboratory diagnosis of acute chikungunya was made by the CDC TRIOPLEX RT-qPCR, which detects CHIKV, dengue (DENV) and Zika viruses (ZIKV) RNA^7^, or by detection of anti-CHIKV IgM antibodies using an ELISA (CHIKjj Detect™ IgM ELISA kits; InBios International, Seattle, WA, USA)^8^.

Telephone interviews were conducted by three trained researchers. To increase the likelihood that patients would respond to telephone calls, they were made at least three times to each potential participant on alternating days and times, including weekends. During the interviews, participants' responses were registered in a database using Research Electronic Data Capture (REDCap) software_._ Participants who reported joint pain for ≥90 days since diagnosis of acute chikungunya were defined as having chronic arthralgia^1^. Conversely, the participants who reported joint pain for <90 days were classified as having no persistent arthralgia. 

To assess general health, we used a self-rated health status question that is widely employed to measure health status^9^ and has previously been correlated with the frequency of chronic pain^10^. This single-item question asks, “In general, how would you say your health is?”. Based on a five-item Likert response scale, we classified the participants as self-reporting as healthy (good, very good, and excellent) or unhealthy (poor and fair). To assess mental health, the Patient Health Questionnaire 9 (PHQ-9) and the Generalized Anxiety Disorder Questionnaire 7 (GAD-7), which are short pre-validated instruments to screen for symptoms of depression and anxiety, respectively, were applied^11^ ^,^ ^12^. Based on the Likert scale responses from the PHQ-9 and GAD-7, participants were classified as having (moderate, moderately severe, or severe) or not having (minimal or mild) symptoms of depression and having (moderate or severe) or not having (minimal or mild) symptoms of anxiety, respectively. For those who reported working when they sought medical care for acute chikungunya, work absenteeism was assessed by asking whether they missed work due to joint pain. Relative and absolute frequencies were calculated to describe the characteristics of the study participants overall and according to arthralgia persistence status. Prevalence ratios (PR) and 95% confidence intervals (95% CI) were calculated to estimate the effects of chronic arthralgia on general health, mental health, and work absenteeism.

All participants provided written informed consent during enrollment in the surveillance study and re-consented orally during the telephone interviews. Participants who reported symptoms during the interview were offered free outpatient consultation at the university rheumatology reference service. This study was approved by the Research Ethics Committee of the Instituto Gonçalo Moniz, Fundação Oswaldo, Cruz (CAAE 55904616.4.0000.0040).

Between June 2019 and March 2020, the surveillance study confirmed 423 autochthonous chikungunya cases using RT-qPCR or IgM detection. Attempts to contact them by telephone were made 20-32 months later and were unsuccessful in 298 (70.4%) patients, mainly because the telephone number was no longer valid, and were successful for 125 (29.6 %) patients . Of the 125 patients whose telephone contact was possible, 63 (50.4%) agreed to be interviewed, and 62 (49.6%) refused. The 63 interviewed participants had a chikungunya diagnosis confirmed by RT-qPCR (38, 60.3%; most of which (30; 78.9%) also had IgM seroconversion or IgM in the acute-phase serum (2; 5.3%)), solely by detection of IgM seroconversion (3, 4.8%), or solely by detection of IgM in the acute-phase sample (22, 34.9%). 

Most (42, 66.7%) of the interviewed patients reported joint pain persistence for ≥90 days and thus were classified as cases of chronic arthralgia. At the time of the interview, 38 (90.5%) of the 42 patients who developed chronic arthralgia persisted with joint pain. Compared to the patients without persistent arthralgia, those reporting chronic arthralgia were more often female (76.2% vs. 57.1%) and aged ≥40 years old (64.3% vs. 42.9%) (Table 1). 

**TABLE 1:** Sociodemographic characteristics of chikungunya patients with and without chronic arthralgia from Salvador, Brazil.

Characteristics	Chikungunya patients with chronic arthralgia	Chikungunya patients without chronic arthralgia
	(N = 42)	(N = 21)
	N (%)	
**Sex**		
Female	32 (76.2)	12 (57.1)
Male	10 (23.8)	9 (42.9)
**Age group, in years**		
18 - 39	15 (35.7)	12 (57.1)
≥40	27 (64.3)	9 (42.9)
**Education**		
Middle school or less	16 (38.1)	6 (28.6)
High school complete or not	22 (52.4)	13 (61.9)
College degree complete or not	5 (11.9)	2 (9.5)
**Working** ^1^		
Yes	34 (82.9)	10 (50.0)
No	7 (17.1)	10 (50.0)
**Laboratory Diagnosis** ^2^		
RT-PCR and IgM seroconversion	19 (45.2)	11 (52.4)
RT-PCR and IgM-positive in acute-phase sample	1 (2.4)	1 (4.8)
RT-PCR only	1 (2.4)	5 (23.8)
IgM seroconversion only	3 (7.1)	0 (0.0)
IgM-positive in the acute-phase sample only	18 (42.9)	4 (19.0)

^1^ Working at the time of acute chikungunya. Data was available for 41 patients with chronic arthralgia and 20 patients without chronic arthralgia. ^2^ A convalescent-phase serum sample was available for 49 chikungunya patients (34 with chronic arthralgia and 15 without chronic arthralgia).

Noteworthy, 68.3% of the participants with chronic arthralgia considered themselves unhealthy, in contrast to 30.0% of those without persistent arthralgia (PR: 2.3; 95% CI: 1.1-4.6) (Table 2). Similarly, depression symptoms were reported by 31.7% and 15.0% of those with and without chronic arthralgia (PR: 2.1; 95% CI: 0.7-6.6), and anxiety symptoms by 43.9% and 35.0%, respectively (PR: 1.3; 95% CI: 0.6-2.5). Absence from work was reported by 76.5% and 40.0% of those with and without chronic arthralgia, respectively (PR, 1.9; 95% CI: 0.9-4.2). 

**TABLE 2:** Prevalence ratios for general health, mental health and absence from work among chikungunya patients with and without chronic arthralgia, Salvador, Brazil.

General health, mental health and work absenteeism	Chikungunya patients with chronic arthralgia ^1^	Chikungunya patients without chronic arthralgia ^1^	Prevalence ratio for health and absenteeism (95% confidence interval)
	(N = 41)	(N = 20)	
	N (%)		N (%)
**General health status** ^2^			
Unhealthy	28 (68.3)	6 (30.0)	2.3 (1.1-4.6)
Healthy	13 (31.7)	14 (70.0)	1.0
**Symptoms of depression** ^3^			
Yes	13 (31.7)	3 (15.0)	2.1 (0.7-6.6)
No	28 (68.3)	17 (85.0)	1.0
**Symptoms of Anxiety** ^4^			
Yes	18 (43.9)	7 (35.0)	1.3 (0.6-2.5)
No	23 (56.1)	13 (65.0)	1.0
**Absence from work** ^5^			
Yes	26 (76.5)	4 (40.0)	1.9 (0.9-4.2)
No	8 (23.5)	6 (60.0)	1.0

^1^ Data available for 41 patients with chronic arthralgia and 20 patients without chronic arthralgia. ^2^ Assessed using the self-rated health status question. Participants who self-reported their general health status as “good”, “very good,” or “excellent” were considered “healthy”. Participants who self-reported their general health status as “poor” or “fair” were considered “unhealthy”. ^3^ Assessed using the Patient Health Questionnaire 9 (PHQ-9). Participants with low PHQ-9 scores (symptoms of depression in the “minimal” or “mild” level) were considered in the “No” group. Participants with high PHQ-9 scores (symptoms of depression in the “moderate”, “moderately severe,” or “severe” level) were considered in the “Yes” group. ^4^ Assessed using the General Anxiety Disorder 7 (GAD-7). Participants with low GAD-7 scores (symptoms of anxiety in the “minimal” or “mild” level) were considered in the “No” group. Participants with high GAD-7 scores (symptoms of anxiety in the “moderate” or “severe” level) were considered in the “Yes” group. ^5^ Only the 44 patients who reported being working answered the question about absenteeism.

These findings reinforce the adverse effects of chronic chikungunya on general and mental health and work attendance, highlighting the need for effective interventions to reduce CHIKV transmission and minimize the health impact of the disease. Furthermore, they corroborate previous studies indicating that chikungunya chronic arthralgia is more common among women and those with increased age. 

Notably, 66.7% of the study participants had chronic arthralgia, which is similar to the findings of other studies^1^. However, this frequency should be interpreted cautiously, as an overestimation is likely because patients who maintained joint pain could be more motivated to participate in the study than those who recovered. This may explain why 90% of the participants with chronic arthralgia still had joint pain at the time of the interview, which was conducted more than one year after the diagnosis of chikungunya. The high frequency of participants who were still symptomatic during the interview may have influenced the negative self-assessment of general and mental health status among the group with chronic arthralgia compared with those without.

Chronic chikungunya pain causes a significant economic burden owing to direct and indirect costs. It also has multidimensional effects, impacting the quality of life in terms of its physical, cognitive, psychoemotional, and sociocultural components, which vastly alters the execution of routine daily activities^13^. In addition, persistent joint pain is commonly accompanied by other symptoms such as insomnia, fatigue, and joint stiffness, contributing to long-term disability and mental suffering^13^. Therefore, patients with acute chikungunya should be warned about the risk of developing chronic symptoms and the need for early multidisciplinary management to minimize the long-term impact of the disease. Care for patients with post-acute and chronic chikungunya should be comprehensive, incorporating physiotherapy, psychological support, and effective pain management provided by well-trained or specialized health professionals. Despite the national public health system's legal provision of free, universal, and integrated care, rehabilitation clinics and trained professionals in Brazil are insufficient to assist these patients. Regional gaps are also enormous, with most health centers located in state capitals and large urban centers. Therefore, significant investment and public health policy development, strengthening, and implementation are required to improve access to care for patients with chronic chikungunya. A broader and stronger healthcare network would benefit not only chikungunya patients but also patients with other chronic diseases, such as mental health illnesses and limiting pain due to other etiologies. 

This study has several limitations. First, we could not reach most of the cases because of the long time between the acute phase of the disease and contact attempts. Furthermore, approximately half of the patients who were contacted refused to be interviewed. The social disruption caused by the COVID-19 pandemic may have contributed to low interest in participating in the study. Although the loss of cases due to failed contact likely did not differ in terms of characteristics between those we reached and those we did not, the loss of cases due to refusal may have introduced bias into our comparisons. As previously mentioned, patients who recovered from joint pain may have been less motivated to participate in the study than those who maintained the pain, potentially leading to an overestimation of the frequency of chronic arthralgia. Conversely, participants whose joint pain lasted more than 90 days but had resolved their symptoms at the time of the interview may have reported having pain for less than 90 days and, therefore, may have been misclassified. 

Second, 34.9% of patients were diagnosed based solely on the detection of anti-CHIKV IgM antibodies in acute-phase samples. Although the possibility that some of these results represent false-positive reactions cannot be ruled out, the specificity of the test in acute-phase samples has been reported to be 97.7%^8^. It is also possible that some of these patients had a prior, recent CHIKV infection (occurring a few months before) instead of an acute infection. 

Third, the use of telephone interviews hampered the possibility of performing a physical examination of the patients to check for pain, edema, or other inflammatory signs in the joints. In addition, as the interviews were conducted during the COVID-19 pandemic, the context may have affected the general and mental health of the study participants. However, any health impact caused by the pandemic must have occurred in both groups, those with and without chronic arthralgia. Thus, it is unlikely that the pandemic can explain the different health levels observed in these groups. 

Finally, if pre-existing joint pathologies were more common among patients with chronic arthralgia, which is possible given that these patients were older and comprised more women than the group without chronic arthralgia, this could have confounded the relationship between chronic joint pain, overall general and mental health, and absenteeism. However, the limited sample size prevented the verification of whether any observed differences were due to potential confounders, including age and sex. Furthermore, the 95% CI for the PR should be interpreted with caution. They help determine the precision of the PR estimates. However, they should not be taken as confidence boundaries for the actual PR in the population from which the study sample was originated because the sample was not randomly selected, violating the norms for the use of statistical inference. Nevertheless, the differences observed in the levels of general health perception, symptoms of depression and anxiety, and work absenteeism were striking and consistent, indicating worse health status among those with chronic arthralgia. 

Our results are in line with those of previous studies, showing that patients with chronic arthralgia have poorer health conditions and higher rates of absenteeism than those without chronic arthralgia^14^. Our results also support the few Brazilian studies that have compared the health consequences of chikungunya according to the development of chronic arthralgia, reinforcing the debilitating effects of the disease on general and mental health and work^3^. The approval of a vaccine against chikungunya in the United States of America in late 2023 brings hope. However, it may take years to gain approval and achieve high coverage in the most affected countries, such as Brazil^15^. Thus, strengthening health services to guarantee the psychological assistance and rehabilitation of patients with chikungunya is urgently needed.

## References

[1] Paixão ES, Rodrigues LC, Costa MDCN, Itaparica M, Barreto F, Gérardin P (2018). Chikungunya chronic disease: a systematic review and meta-analysis. Trans R Soc Trop Med Hyg.

[2] Doran C, Duits A, Tami A, Gerstenbluth I, Bailey A (2023). "It's very saddening, you keep on wondering when the symptoms will be over": A qualitative study exploring the long-term chikungunya disease impact on daily life and well-being, 6 years after disease onset. PLoS Negl Trop Dis.

[3] Silva MMO, Kikuti M, Anjos RO, Portilho MM, Santos V, Gonçalves TSF (2021). Risk of chronic arthralgia and impact of pain on daily activities in a cohort of patients with chikungunya virus infection from Brazil. Int J Infect Dis.

[4] Simon F, Bossy R, Federico D, Dezaunay J, Demoux AL, Rugard N (2022). Determinants of Health-Related Quality of Life in Chronic Chikungunya Disease in Guadeloupe. Pathogens.

[5] Marimoutou C, Vivier E, Oliver M, Boutin JP, Simon F (2012). Morbidity and impaired quality of life 30 months after chikungunya infection: comparative cohort of infected and uninfected French military policemen in Reunion Island. Medicine.

[6] Ramachandran V, Malaisamy M, Ponnaiah M, Kaliaperuaml K, Vadivoo S, Gupte MD (2012). Impact of Chikungunya on health related quality of life Chennai, South India. PLoS ONE.

[7] Santiago GA, Vázquez J, Courtney S, Matías KY, Andersen LE, Colón C (2018). Performance of the Trioplex Real-Time RT-PCR Assay for Detection of Zika, Dengue, and Chikungunya Viruses. Nat Commun.

[8] Kikuti M, Tauro LB, Moreira PSS, Nascimento LCJ, Portilho MM, Soares GC (2020). Evaluation of two commercially available chikungunya virus IgM enzyme-linked immunoassays (ELISA) in a setting of concomitant transmission of chikungunya, dengue and Zika viruses. Int J Infect Dis.

[9] Bombak AE (2013). Self-rated health and public health: a critical perspective. Front Public Health.

[10] Mäntyselkä PT, Turunen JH, Ahonen RS, Kumpusalo EA (2003). Chronic pain and poor self-rated health. JAMA.

[11] Santos SI, Tavares FB, Munhoz NT, Almeida PSL, Silva BTN, Tams DB (2013). Sensitivity and Specificity of the Patient Health Questionnaire-9 (PHQ-9) among adults in the general population. Cad Saude Publica.

[12] Silva SL, Leite FM, Feitosa BLA, Faro A (2023). Psychometric properties of GAD-7 in Brazil School of Health and Life Sciences. Psico.

[13] Van Aalst M, Nelen CM, Goorhuis A, Stijnis C, Grobusch MP (2017). Long-term sequelae of chikungunya virus disease: A systematic review. Travel Med Infect Dis.

[14] Couzigou B, Criquet-Hayot A, Javelle E, Tignac S, Mota E, Rigaud F (2018). Occurrence of Chronic Stage Chikungunya in the General Population of Martinique during the First 2014 Epidemic: A Prospective Epidemiological Study. Am J Trop Med Hyg.

[15] De Souza WM, Ribeiro GS, de Lima STS, de Jesus R, Moreira FRR, Whittaker C (2024). Chikungunya: a decade of burden in the Americas. Lancet Reg Health Am.

